# The association of urinary phosphorous-containing flame retardant metabolites and self-reported personal care and household product use among couples seeking fertility treatment

**DOI:** 10.1038/s41370-019-0122-9

**Published:** 2019-02-06

**Authors:** Mary E. Ingle, Lidia Mínguez-Alarcón, Courtney C. Carignan, Craig M. Butt, Heather M. Stapleton, Paige L. Williams, Jennifer B. Ford, Russ Hauser, John D. Meeker, for the EARTH Study Team

**Affiliations:** 1 Department of Environmental Health Sciences, University of Michigan School of Public Health, Ann Arbor, MI USA; 2 Department of Environmental Health, Harvard T.H. Chan School of Public Health, Boston, MA USA; 3 Department of Food Science and Nutrition, Michigan State University, East Lansing, MA USA; 4 Department of Pharmacology and Toxicology, Michigan State University, East Lansing, MI USA; 5 Nicholas School of the Environment, Duke University, Durham, NC USA; 6 Department of Biostatistics, Harvard T.H. Chan School of Public Health, Boston, MA USA; 7 Department of Epidemiology, Harvard T.H. Chan School of Public Health, Boston, MA USA; 8 Obstetrics and Gynecology, Massachusetts General Hospital, Harvard Medical School, Boston, MA USA

## Abstract

**Background:**

Phosphorous-containing flame-retardants (PFRs) are widely detected. They are used both as a flame retardant as well as plasticizer.

**Methods:**

A subset of 230 women and 229 men were recruited from Massachusetts General Hospital fertility clinic between 2005 and 2015. At each visit, participants completed a questionnaire of personal care product (PCP) and household product (HP) use. Metabolites [bis(1,3-dichloro-2-propyl) phosphate, diphenyl phosphate (DPHP), isopropylphenyl phenyl phosphate (ip-PPP), tert-butylphenyl phenyl phosphate and bis(1-chloro-2-propyl) phosphate] were measured in urine (1–5 samples; *n* = 638 women, *n* = 335 men). Associations were assessed using generalized mixed models, adjusted for SG, age, BMI, smoking, education, and season.

**Results:**

In women, moisturizer (60%), nail polish remover (77%), and nail polish (134%) use were associated (*p* < 0.05) with an increase in DPHP concentrations, while ip-PPP concentrations increased 21–27% with conditioner, cosmetics, deodorant, and hair product use. Mouthwash and vinyl glove use were associated with a respective 31% and 92% increase in DPHP among men.

**Conclusions:**

Our exploratory analysis suggests PFRs may be used as a plasticizer in consumer products, and nail polish use contributes to internal DPHP exposure. Further research is needed to understand how PFRs are used in these products and how it relates to exposure.

## Introduction

Organophosphate esters have been used as flame retardants (FR) for over 150 years [1, 2]. The use of phosphorous-containing FRs (PFRs) has grown drastically since the phase out of polybrominated diphenyl ethers (PBDEs) in the past decade due to concerns regarding their persistence and toxicity [3–5]. As their prevalence rose, PFRs evolved into a high production volume chemical with U.S. production projected to reach approximately 50,000 tons per year by 2020 for certain compounds [6]. PFRs include both chlorinated alkyl esters such as tris(2-chloroisopropyl) phosphate and tris(1,3-dichloroisopropyl) phosphate (TDCIPP), and nonhalogenated aryl phosphates such as triphenyl phosphate (TPHP) and isopropyl triphenyl phosphate (ITP) [7]. TPHP and ITP account for 60% of Firemaster^®^ 550, a widely used commercial flame retardant mixture that replaced PBDEs in furniture foams and baby products, yet are also used as a plasticizer in paints, lacquers, and varnishes [8–11].

Considered “additive” compounds, PFRs are physically added or “mixed” with materials during manufacturing, rather than being chemically bound [12, 13]. The main route of exposure of PFRs was thought to be dust ingestion as a result of the weak bonds allowing for volatilization and settlement into dust of indoor environments [14, 15]. However, recent studies using air samplers, hand wipes and silicone wrist bands have shown that inhalation and dermal exposure may also be pathways of exposure [6, 11, 16]. Despite the short biological half-lives ranging from a few hours to days, metabolites of PFRs have been detected in nearly 100% of urine samples among women, men, and children in the U.S. and Europe [17–21]. Despite being rapidly metabolized once in the body, high detection of PFRs suggests exposure is continuous and widespread. Research on the health effects of PFRs is limited, although prior studies have shown adverse immunologic and neurologic outcomes, as well as associations with the disruption of endocrine, reproductive, and developmental systems [22–26]. As of 2011, TDCIPP and tris(2-chloroethyl) phosphate are listed as a known carcinogens by the state of California [27].

These compounds have been highly detected in environmental samples, and primary sources are thought to be polyurethane foams found in furniture, baby products, and electronics [28–30]. Few studies to date have assessed the prevalence of PFRs in other consumer and personal care products (PCP) where they may be utilized as a plasticizer. A widely used PFR, TPHP is commonly listed as an ingredient in nail polishes. Several studies have also detected TPHP in products where it was not listed as an ingredient [31–33]. A small study found urine concentrations of diphenyl phosphate (DPHP), a metabolite of TPHP, to increase sevenfold after nail polish application [32]. Our present work expands upon this preliminary evidence to characterize the relationship between self-reported PCP and household product (HP) use within 24 h. of a urine sample measuring the concentration of five PFR metabolites: bis(1-chloro-2-propyl) phosphate (BCIPP), bis(1,3-dichloro-2-propyl) phosphate (BDCIPP), DPHP, isopropylphenyl phenyl phosphate (ip-PPP), tert-butylphenyl phenyl phosphate (tb-PPP) among couples attending a fertility clinic.

## Materials and methods

### Participant recruitment

Couples from this analysis are a subset from the Environment and Reproductive Health (EARTH) Study, an existing prospective cohort assessing the impact of environmental agents on reproductive health. Recruitment and participation have been previously described [25, 34]. Briefly, women (18–46 y) and men (18–55 y) were recruited from Massachusetts General Hospital (MGH) Fertility Center between 2005 and 2015. Among couples approached for the EARTH study, approximately 60% of women and 50% of men agreed to participate. Women were included in this analysis if they provided at least one urine sample for PFR measurement and completed the PCP and HP questionnaire during an in vitro fertilization cycle, while men must have provided at least one urine sample for PFR measurement, completed the questionnaire, and have a woman partner also in the study [35]. Men were excluded only if they had a prior vasectomy. Informed consent was signed by each participant and Institutional Review Board approval was received by all institutions.

### PCP and HP questionnaires

At the time of enrollment, couples completed questionnaires capturing demographic, health history, and lifestyle factors. At the beginning of each subsequent visit, women and men completed a questionnaire on PCP (*n* = 20 products) and HP (*n* = 14 products) use within the last 24 h. Consumer products with *n* < 5 participants reporting use in the last 24 h were excluded from the analysis.

### Urine collection and PFR analysis

One urine sample (up to five samples per participant) was collected in sterile polypropylene cups from couples at each visit. After collection, specific gravity (SG) was measured for each sample using a Protometer handheld 100B refractometer (National Instrument Company, Inc., Austin, TX). Samples were then separated into aliquots and frozen (−80°) prior to overnight shipment on dry ice to H.M. Stapleton’s laboratory at Duke University (Durham, NC) for analysis.

Extraction and analysis of PFR metabolites BCIPP, BDCIPP, DPHP, ip-PPP, and tb-PPP have been established and previously described [36]. Briefly, samples were thawed and separated into glass tubes in 5 mL aliquots and spiked with internal standards (d_10_-BDCIPP = 80 ng, d_10_-DPHP = 60 ng). Samples were then acidified to pH < 6.5 with formic acid and diluted 1:1 with water. Samples were concentrated and cleaned using solid phase extraction before nitrogen stream drying and then spiked with recovery standard (^13^C_2_-DPHP = 81.5 ng). Extracts were analyzed using negative electrospray ionization liquid chromatography tandem mass spectrometry (LC-MS/MS). Data were acquired using optimal parameters under multiple reaction conditions. Internal standard used for BCIPP and BDCIPP was d_10_-BDCIPP, while DPHP, ip-DPHP, and tb-PPP were quantified using d_10_-DPHP. Urinary SG ranged from 1.002 to 1.100 (geometric mean (GM) = 1.104) for women and 1.011 to 1.038 (GM = 1.017) for men.

Procedures for quality control and assurance for LC-MS/MS have been previously reported [25]. Samples were analyzed in ten separate batches including five blanks (5 mL Mili-Q water) to establish a distinct method detection limit (MDL) for each batch. Laboratory blanks were multiplied three times the standard deviation to establish MDLs which ranged from 0.07 to 0.17 pg/mL for BCIPP, 0.02 to 0.11 pg/mL for BDCIPP, 0.09 to 0.18 pg/mL for DPHP, 0.06 to 0.12 pg/mL for ip-PPP, and 0.04 to 0.15 pg/mL for tb-PPP. A standard reference material was established using pooled samples from prior studies and precision was evaluated with duplicates of two subsamples.

### Statistical analysis

Distributions of PFRs have been previously reported for participants [25, 37]. Due to the low frequency of detectable concentrations, BCIPP was henceforth excluded in this analysis. Metabolite concentrations below MDL were imputed as MDL/√2. An aggregate variable (∑PFR) was imputed by summing the molar urinary concentrations for metabolites BDCIPP, DPHP, and ip-PPP. Spearman correlation coefficients were calculated for each metabolite among 229 couples. Metabolites BDCIPP, DPHP, ip-PPP, and ∑PFR presented as right-skewed and were therefore transformed by the natural logarithm for further statistical modeling.

**Fig. 1:**
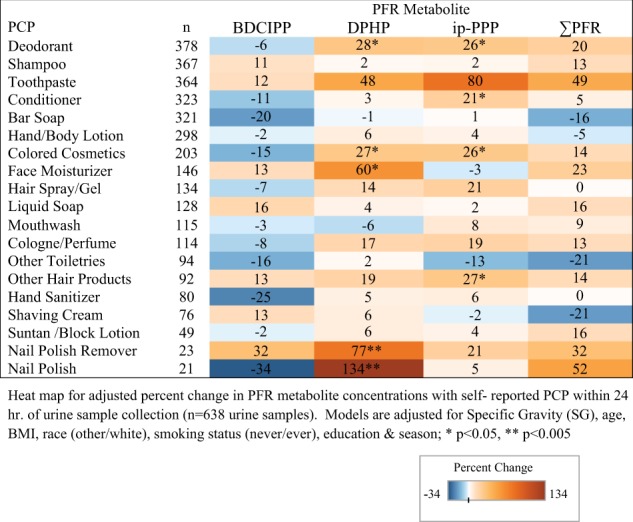
Adjusted percent change in urinary PFR metabolite concentrations with self-reported PCP use for 230 women from the EARTH cohort

**Fig. 2:**
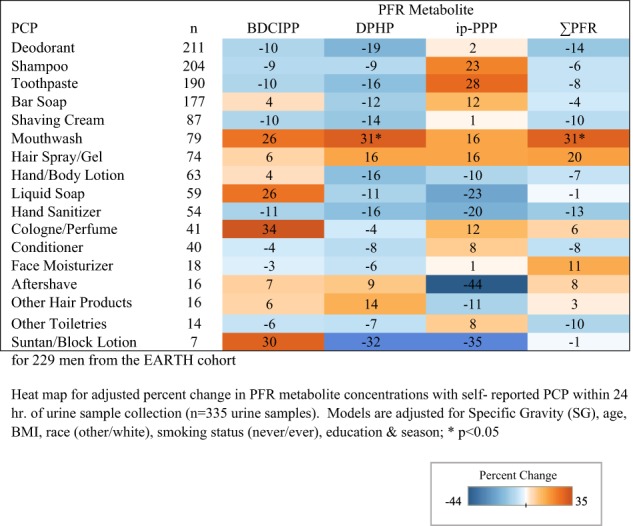
Adjusted percent change in urinary PFR metabolite concentrations with self-reported PCP use

**Fig. 3:**
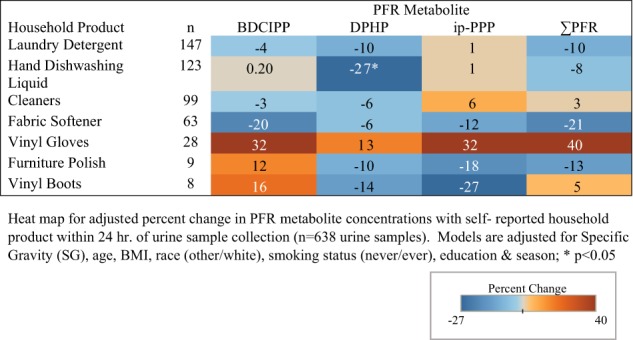
Adjusted percent change in urinary PFR metabolite concentrations with self-reported household product use for 230 women from the EARTH cohort

**Fig. 4:**
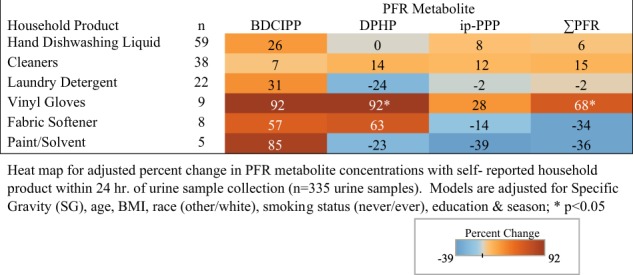
Adjusted percent change in urinary PFR metabolite concentrations with self-reported household product use for 229 men from the EARTH cohort

Questionnaire responses to PCP and HP use within the last 24 h was evaluated as binary (“yes” or “no”) where those who responded “don’t know” (*n* < 9 per consumer product) were recoded as a “no” response. PFR metabolites were evaluated as continuous variables except for tb-PPP, which had low-detection rates (13.32% for women and 11.34% for men), and was modeled as detect/nondetect (data not shown). Covariates for modeling were selected a priori and through bivariate testing (data not shown) [34, 38, 39]. Final models were adjusted for SG, age, BMI, race (other/Caucasian) smoking (never/ever), education (high school, some college/college, or graduate/graduate degree), and season (winter/spring/summer/fall). Models that included year of collection had decreased goodness of fit and are not presented. Missing covariates were imputed with the median for continuous variables (age = 34 for women, *n* = 16 and BMI = 26.84 for men, *n* = 3) and the category with the highest frequency (education = graduate degree, *n* = 61 women and *n* = 38 men). Multivariable generalized mixed models were used to evaluate associations with repeated PCP and HP use (exposure) and PFR metabolite concentrations (outcome) using a normal distribution with identity link (Supplemental Tables 1–4). Regression coefficients and 95% confidence intervals (CI) were transformed to reflect the adjusted percent change in urinary PFR metabolite concentrations with reported use of each PCP and HP within 24 h. of urine sample (Supplemental Tables 5–8). Heat maps were then generated to graphically display the adjusted percent change (Figs. 1–4; statistical significance is indicated by an asterisks on maps). A sensitivity analysis dividing observations into 5 year increments (2005–2009 and 2010–2015) was conducted to investigate possible changes in consumer product formulations by time period (data not shown). All statistical analyses were carried out using SAS 9.4 (SAS Institute Inc., Cary, NC).

## Results

Our subset from the EARTH cohort consisted of 230 women and 229 men contributing one to five urine samples per participant (*n* = 638 women and *n* = 335 men). Demographic characteristics of these women and men have been previously reported [25, 37]. Briefly, this sample consisted primarily of Caucasian (87%), nonsmoking (75%), and highly educated (57% hold graduate degree) women with an average age of 35 [25]. Men were slightly older than women (mean = 36.78 y), yet also predominantly Caucasian (89%), non-mokers (70%) with 82% holding a college degree or higher [37].

Self-reported PCP use for women and men within 24 h of urine sample collection are depicted in Table 1. The most commonly used PCPs by women were deodorant (*n* = 378), shampoo (*n* = 367), toothpaste (*n* = 364), conditioner/cream rinse (*n* = 323), and bar soap (*n* = 321). Suntan/sun block lotion (*n* = 49), nail polish remover (*n* = 23), and nail polish (*n* = 21) had the lowest reported use. Similarly, men also frequently reported using deodorant (*n* = 211), shampoo (*n* = 204), toothpaste (*n* = 190), and bar soap (*n* = 177), but also shaving cream (*n* = 87). Less frequently reported PCPs among men included aftershave, other hair products, and suntan/sunblock lotion (<10%). Reported HP use was less frequent among women and men compared to PCP (Table 2). However, both women and men reported use of laundry detergent, hand dishwashing liquid and cleaners. For women, vinyl gloves, furniture polish, and vinyl boots were less frequently used (<6%), while vinyl gloves, fabric softener, and paint/solvents were the least reported HP for men (<5%).

**Table 1:** Summary of reported personal care product use within 24 h of PFR urine sample collection among EARTH participants between 2005 and 2015 (*n* = 230 women; *n* = 229 men)

Personal care product	Yes (*n*, %)	No (*n*, %)	Missing (*n*, %)
*Women (*n *=* *638 samples)*			
Deodorant	(378, 59.2)	(101, 15.8)	(159, 24.9)
Shampoo	(367, 57.5)	(112, 17.6)	(159, 24.9)
Toothpaste	(364, 57.1)	(3, 0.4)	(271, 42.5)
Conditioner/cream rinse	(323, 50.6)	(156, 24.5)	(159, 24.9)
Bar soap	(321, 50.31)	(157, 24.6)	(160, 25.1)
Hand/body lotion	(298, 46.8)	(181, 28.4)	(159, 24.9)
Colored cosmetics	(203, 31.8)	(276, 43.3)	(159, 24.9)
Face moisturizer/lotion	(146, 22.9)	(37, 5.8)	(455, 71.3)
Hair spray/hair gel	(134, 21.0)	(341, 53.4)	(163, 25.5)
Liquid soap/body wash	(128, 20.1)	(55, 8.6)	(455, 71.3)
Mouthwash	(115, 18.0)	(349, 54.7)	(174, 27.3)
Cologne or perfume	(114, 17.9)	(365, 57.2)	(159, 24.9)
Other toiletries	(94, 14.7)	(54, 8.4)	(490, 76.8)
Other hair products	(92, 14.4)	(386, 60.5)	(160, 25.1)
Hand sanitizer	(80, 12.5)	(286, 44.8)	(272, 42.6)
Shaving cream	(76, 11.9)	(403, 63.2)	(159, 24.9)
Suntan/sunblock lotion	(49, 7.7)	(430, 67.4)	(159, 24.9)
Nail polish remover	(23, 3.6)	(159, 24.9)	(456, 71.5)
Nail polish	(21, 3.3)	(457, 71.6)	(160, 25.1)
*Men (*n = *335 samples)*			
Deodorant	(211, 63.0)	(37, 11.0)	(87, 26.0)
Shampoo	(204, 60.9)	(44, 13.1)	(87, 26.0)
Toothpaste	(190, 56.7)	(4, 1.2)	(141, 42.1)
Bar soap	(177, 52.8)	(71, 21.2)	(87, 26.0)
Shaving cream	(87, 13.6)	(161, 43.8)	(87, 26.0)
Mouthwash	(79, 23.6)	(169, 50.4)	(87, 26.0)
Hair spray/hair gel	(74, 22.1)	(174,51.9)	(87, 26.0)
Hand/body lotion	(63, 18.8)	(184, 54.9)	(88, 26.3)
Liquid soap/body wash	(59, 17.6)	(48, 14.3)	(228, 68.1)
Hand sanitizer	(54, 16.1)	(139, 41.5)	(142, 42.4)
Cologne or perfume	(41, 12.2)	(206, 61.5)	(88, 26.3)
Conditioner/cream rinse	(40, 11.9)	(208, 62.1)	(87, 26.0)
Face moisturizer/lotion	(18, 5.4)	(90, 26.9)	(227, 67.8)
Aftershave	(16, 4.8)	(232, 69.3)	(87, 26.0)
Other hair products	(16, 4.8)	(230, 68.7)	(89, 26.6)
Other toiletries	(14, 4.18)	(77, 23.0)	(244, 72.8)
Suntan/sunblock lotion	(7, 2.1)	(241, 71.9)	(87, 26.0)

Other hair products include: mousse, hair bleach, relaxer, perm straightener; other

toiletries include: wax, vaseline, lip balm; reported use of personal care products *n* < 5 were not listed

**Table 2:** Summary of reported household product use within 24 h of PFR urine sample among EARTH participants between 2005 and 2015 (*n* = 230 women; *n* = 229 men)

Household product	Yes (*n*, %)	No (*n*, %)	Missing (*n*, %)
*Women (*n *=* *638)*			
Laundry detergent	(147, 23.1)	(332, 52.3)	(159, 24.9)
Hand dishwashing liquid	(123, 19.3)	(60, 9.4)	(455, 71.3)
Cleaners	(99, 15.5)	(380, 59.6)	(159, 24.9)
Fabric softener	(63, 9.9)	(416, 65.2)	(159, 24.9)
Vinyl gloves	(28, 4.4)	(451, 70.7)	(159, 24.9)
Furniture polish	(9, 1.4)	(470, 73.7)	(159, 24.9)
Vinyl boots	(8, 1.3)	(471, 73.8)	(159, 24.9)
*Men (*n *=* *335)*			
Hand dishwashing liquid	(59, 17.6)	(49, 14.6)	(227, 67.8)
Cleaners	(38, 11.3)	(210, 62.7)	(87, 26.0)
Laundry detergent	(22, 6.6)	(226, 67.5)	(87, 26.0)
Vinyl gloves	(9, 2.7)	(238, 71.0)	(88, 26.3)
Fabric softener	(8, 2.4)	(240, 7.2)	(87, 26.0)
Paint/solvents	(5, 1.5)	(103, 30.7)	(227, 67.8)

Reported use of household products *n* < 5 were not listed

Metabolites BDCIPP, DPHP, and ip-PPP were highly detected among both women (85%, 90%, and 75%) and men (85%, 86%, and 67%) [25, 37]. Metabolite concentrations were higher among women compared to men for BDCIPP and DPHP, similar for ip-PPP, while tb-PPP concentrations were higher in men. Metabolite concentration correlations among couples (*n* = 229) were weak for all metabolites (0.20 < *r* < 0.31, *p* ≤ 0.01), with the exception of tb-PPP (*r* = 0.70, *p* = 0.04), that was detected at low rates (<15% above MDL) for both women and men (Table 3). Concentrations of DPHP for men were also weakly correlated to ip-PPP concentrations in women (*r* = 0.14, *p* = 0.04).

**Table 3:** Spearman correlation coefficients of urinary PFR metabolites among couples in the EARTH cohort (*n* = 229 couples)

		Women PFR metabolites							
		BDCIPP		DPHP		ip-PPP		tb-DPHP	
		*r*	*p* Value	*r*	*p* Value	*r*	*p* Value	*r*	*p* Value
Men PFR metabolites	BDCIPP	**0.31**	<0.0001	0.03	0.58	0.06	0.41	−0.26	0.11
	DPHP	0.06	0.33	**0.22**	0.0002	**0.14**	0.04	−0.05	0.76
	ip-PPP	0.06	0.42	0.06	0.43	**0.20**	0.01	−0.05	0.81
	tb-DPHP	0.02	0.92	0.09	0.56	0.15	0.44	**0.70**	0.04

Bold values represent correlation coefficients with a *p* < 0.05

Adjusted percent change in PFR metabolite concentrations with self-reported PCP use for women can be found in Fig. 1. Use of nail polish was associated with a 134% increase (95% CI: 62–235; *p* < 0.0001) in DPHP concentrations as well as nail polish remover (77%; 95% CI: 21–159; *p* = 0.0004). Increased concentrations of DPHP were associated with reported use of face moisturizer (60%; 95% CI: 15–123; *p* = 0.01). Deodorant use was also associated with a 28% increase in DPHP (95% CI: 5–57; *p* = 0.02) and a 26% increase in ip-PPP (95% CI: 1–55; *p* = 0.04) concentrations. We identified significant relationships with the use of colored cosmetics with DPHP and ip-PPP concentrations (27%, 95% CI: 7–49; *p* = 0.01 and 26%, 95% CI: 1–55; *p* = 0.01, respectively). Reported use of hair products including mousse, hair bleach, relaxer, and perm straightener was associated with a 27% increase (95% CI: 2–58; *p* = 0.04) in ip-PPP concentrations, while toothpaste was associated with a nonsignificant increase of 80% (95% CI: −35 to 395; *p* = 0.25). Yet for men, only mouthwash was significantly associated with a 31% increase in DPHP (95% CI: 3–63; *p* = 0.03) and total PFR (95% CI: 8–62; *p* = 0.01) concentrations (Fig. 2).

Vinyl glove use was associated with a 32% increase in BDCIPP and ip-PPP concentrations among women (95% CI: −20 to 120; *p* = 0.27 and 95% CI: −11 to 97; *p* = 0.17), while concentrations of DPHP were related to a 27% decrease (95% CI: −45 to −3; *p* = 0.03) with reported hand dishwashing liquid use (Fig. 3). Vinyl glove use in men had the highest associated increase in BDCIPP (95% CI: −12 to 314; *p* = 0.10), DPHP (95% CI: 9–232; *p* = 0.02), and total PFR (95% CI: 3–175; *p* = 0.04) concentrations (92%, 92%, and 68%, respectively) (Fig. 4). While not statistically significant, concentrations of BDCIPP were associated with an 85% increase with the use of paints/solvents (95% CI: −73 to 169; *p* = 0.77), while fabric softener use was related to an 57% increase in BDCIPP (95% CI: −74 to 25; *p* = 0.16) and 63% increase in DPHP (95% CI: −65 to 12; *p* = 11) concentrations.

In our sensitivity analysis, nail polish use was associated with a 306% increase in urinary DPHP concentrations (95% CI: 129–610; *p* < 0.0001) among women with samples collected between 2010 and 2015. Metabolite concentrations of ip-PPP in women increased by 40% in association with deodorant use during 2010–2015 (95% CI: 5–88; *p* = 0.02). All significant associations with PFRs and PCP and HP highlighted in our primary analysis remained the same or similar when only using observations collected during 2010 or later. Although, these associations disappeared when only using observations between 2005 and 2009 (data not shown). No other significant associations were identified during this secondary analysis.

## Discussion

We identified several associations with PCP use and DPHP concentrations, specifically with the use of nail polish, nail polish remover, face moisturizer, deodorant, and cosmetic use in women. Concentrations of ip-PPP were also associated with increased reported use of deodorant, cosmetics, and hair products. We did not observe similar relationships with PCP use in men, only finding associations with DPHP and total PFR with mouthwash. Overall, there were few significant associations identified with HP use. Reported use of vinyl gloves was associated with elevated concentrations of all metabolites between men and women. Although most relationships were associated with an increase in PFR metabolite concentration, dishwashing liquid use was significantly associated with a decrease in DPHP concentrations among women.

We found PFR concentrations to be slightly higher in women compared to men for BDCIPP and DPHP in this study. The National Health and Nutrition Examination Survey (NHANES) reported similar results with slightly higher concentrations of DPHP in women (GM = 0.92 μg/L) compared to men (GM = 0.78 μg/L), yet concentrations of BDCIPP were slightly higher for men (GM = 0.91 μg/L) compared to women (GM = 0.80 μg/L) [3]. Concentrations of DPHP for men and women from NHANES were similar to our sample (women GM = 0.91, men GM = 0.75 μg/L), though BDCIPP metabolite concentrations were slightly higher for women (GM = 0.91 μg/L) and lower for men (GM = 0.62 μg/L). A small study (*n* = 53) in North Carolina found a slightly larger sex disproportion of DPHP with women having approximately a twofold higher urinary concentration (10^β^ = 1.84) compared to men (10^β^ = 0.98), although BDCIPP concentrations were comparable [12]. Similar to our findings, phthalate metabolites have also been found at higher concentrations in women compared to men, which are also used as a plasticizer in various PCP and HP products [40, 41].

Along with the differences in PFR concentrations by sex, the lack of similar relationships between self-reported PCP use and PFR metabolite concentrations could possibly be a result of the episodic use patterns of PCPs, lifestyle, as well as different formulations of products targeted to each sex [3, 35]. A U.S. survey of 2300 adults found the average women uses 12 products consisting of approximately 168 unique ingredients per day while men use an average of 6, exposing them to 85 unique chemicals in a single day [42]. Higher reported usage of both PCP and HP among women have also been reported in studies from the Netherlands, Switzerland, and South Korea [43–45].

Organophosphates have been largely associated with their use as FRs in polyurethane foam in furniture and cars, electronics, as well as components of the widely used FR mixture Firemaster^®^ 550 [30, 46–48]. However, nonhalogenated compounds like TPHP and ITP are also used as plasticizers [8]. Plasticizers are frequently used to increase the flexibility plastics and in the production of vinyl in PCP and HP [49, 50]. This coincides with the majority of our relationships identified with increasing concentrations of TPHP and ITP metabolites (DPHP and ip-PPP, respectively) with reported use of PCP and HP. TPHP is commonly listed as an ingredient in nail polishes where it is likely used to increase the flexibility of the polish after its application. A small study from the California Environmental Protection Agency (Cal EPA) detected TPHP in five of 14 nail products tested [31]. Interestingly, when TPHP was detected, a common plasticizer, dibutyl phthalate (DBP) was not found. Thus, TPHP is possibly replacing DBP in nail products as a plasticizer. This potentially explains our strongest association of percent increase for DPHP concentrations (134%) with reported nail polish use which more than doubled (306%) when only using observations collected during 2010 or later. Our findings also overlap with a prior study of urinary DPHP concentrations and nail polish application that found a larger increase in urine concentrations (sevenfold) compared to our results, which could be a result of more rigorous and timely urine collection in their study design [32]. We also detected a significant association with nail polish remover and elevated DPHP concentrations (77%). This result was unexpected as acetone, ethylene glycol, and gamma butyrolactone are the most common ingredients for nail polish remover [51]. While this could be a result of PFRs in the product, it is also possible that as nail polish is being removed, exposure via inhalation or dermal absorption is increased. Or perhaps nail polish remover could be acting as a surrogate for nail polish as use of both products was correlated (*p* = 0.001). Deodorant use among women was also correlated with nail polish use (*p* = 0.02) and surrogacy possibly explains this unexpected result. In our secondary analysis we also observed a 40% increase in ip-PPP urine concentrations with observations between 2010 and 2015 (compared to a 20% increase using all observations) for women who reported using deodorant. Nail polish and deodorant were the only products to have a substantial difference in PFR concentrations when exploring the year of collection. Along with TPHP replacing DBP as a plasticizer in nail polish, it is also possible that phthalates used in deodorants are also being replaced with PFRS like TPHP or ITP [52]. These results further highlight the possibility of PFRs replacing phthalates as plasticizers around 2010 [33].

Interestingly, we also observed a significant decrease in DPHP concentrations with reported dishwashing liquid use. This is possibly due to reduced dermal absorption as a result of frequent hand washing which has been reported for several PFRs [53]. Although not significant, we also observed decreased PFR concentrations with reported conditioner and bar soap use in women and shampoo, toothpaste, and shaving cream use among men, which could also be a result of washing, rinsing, or bathing to decrease dermal absorption.

Weak correlations of metabolites among couples suggest that dust ingestion from PFRs in furniture foams from the home is not the sole exposure route/pathway. Organophosphate esters are characterized as semi volatile organic compounds and continuously divided between the gaseous and solid phases, thus exposure to PFRs is possibly from multiple routes/pathways causing them to be ingested as well as absorbed through the skin from PCP and HP use [54]. These weak correlations could be a result of differences in PCP and HP use patterns between sexes, or varying metabolism rates of PFRs among women and men.

Although novel, our study was subject to several limitations. The PCP and HP questionnaire did not capture frequency, amount, or the last time of product use in relation to urine sample collection. However, due to the exploratory nature of this study, additional adjustment for these factors may have saturated our model and biased our results toward the null. PFR exposure differences among couples could be a result of being in different environments throughout the day as PFRs have also been highly detected in cars and offices [7]. Despite being a comprehensive PCP and HP use questionnaire, our results may be susceptible to a factor of multiple statistical comparisons. Nevertheless, our results coincide with the prior studies of DPHP and nail polish [32].

There is also a possibility that several factors may have minimized our findings. Due to the rapid metabolism of PFRs, resulting in half-lives of several hours, as well as the episodic nature of PCP and HP use, it is possible our effect sizes of our relationships are underestimated [18, 35]. Although, studies from the same cohort found moderate temporal variability among PFR concentrations over a 3 month time period [18, 25]. While our study consisted of approximately 230 couples, we had almost twice as many women urine samples (*n* = 638) compared to men (*n* = 335). A difference in repeated measurements could explain the sex differences we observed in our relationships with PCP and HP. PFR concentrations in women have also been found to be higher compared to men, including our present findings [3]. Our study population was comprised primarily of Caucasian, nonsmoking, highly educated couples who were subfertile, which could also have resulted in modest results as prior studies have found higher PFR concentrations associated with lower socioeconomic status and non-Caucasian populations [3, 21]. Thus, our results may only be generalizable to similar populations, yet identify the necessity to investigate these associations in diverse populations.

Our study also had several strengths. To the best of our knowledge, we are the most comprehensive study to date to assess the potential relationships with PFR metabolite concentrations with self-reported PCP and HP use. Our study design also allowed for increased precision of PFR metabolites due to multiple urine samples as well as multiple questionnaire responses per participant. The prospective study design also decreased the possibility of systematic error as the questionnaire referenced product use only 24 h prior to urine sample collection. Also, as our sample consisted of couples, we were able to identify the lack of correlation between metabolites among couples likely residing in the same residence to exploit the possibility for alternate exposure routes and pathways besides dust from the home. Finally, the longevity of this well-established cohort spanning 10 years of sample collection allowed for the exploration of temporal associations correlating to formulation changes of PCP and HPs.

## Conclusion

To the best of our knowledge, this is the first study to characterize the relationships of PFR metabolites and self-reported PCP and HP use. While metabolites BDCIPP, DPHP, and ip-PPP were highly detected among women and men, concentrations in women were slightly higher. Correlations of metabolites were weak among couples which is consistent with our different results among sexes. Similar to the only prior study of DPHP in urine and nail polish, we identified an association of increased DPHP concentrations in urine (134%) with reported nail polish use. This association nearly doubled (306% increase) when only using observations from 2010 to 2015. We also identified significant associations with reported use of other PCPs including: nail polish remover, face moisturizer, colored cosmetics, and deodorant for women. Relationships with HP use were fewer, yet vinyl glove use in men was associated with a 92% increase in DPHP concentrations. These results suggest PFRs are not only used as FRs, but possibly as plasticizers in these products and also contribute to internal exposure. Furthermore, it is possible this replacement occurred during 2010 as highlighted by our differences in associations using observations between 2005–2009 compared to those between 2010 and 2015. Our results identify the necessity of more targeted studies to further investigate the prevalence of PFR compounds, especially non-halogenated aryl phosphates TPHP and ITP, in PCPs and HPs highlighted by our results.

## Supplementary information

Supplementary Information
