# Supplementary material for: The association of urinary phosphorous-containing flame retardant metabolites and self-reported personal care and household product use among couples seeking fertility treatment
Source: J Expo Sci Environ Epidemiol. 2019 Feb 6;30(1):107–16. doi: 10.1038/s41370-019-0122-9 (PMC6914666; doi:10.1038/s41370-019-0122-9)
Supplement: Supplementary file 1 — Supplementary Information [file 41370_2019_122_MOESM1_ESM.docx]

The association of urinary phosphorous-containing flame retardant metabolites and self-reported personal care and household product use among couples seeking fertility treatment

Supplementary Information

Mary E. Ingle ^a^, Lidia Mínguez-Alarcón ^b^, Courtney C. Carignan ^c, d^, Craig M. Butt ^e^, Heather M. Stapleton ^e^, Paige L. Williams ^f, g^, Jennifer B. Ford ^b^, Russ Hauser ^b, g, h^, John D. Meeker ^a, *^, for the EARTH Study Team

^a^ Department of Environmental Health Sciences, University of Michigan School of Public Health, Ann Arbor, Michigan, USA

^b^ Department of Environmental Health, Harvard T.H. Chan School of Public Health, Boston, Massachusetts, USA

^c^ Department of Food Science and Nutrition, Michigan State University, East Lansing, Michigan, USA

^d^ Department of Pharmacology and Toxicology, Michigan State University, East Lansing, Michigan, USA

^e^ Nicholas School of the Environment, Duke University, Durham, North Carolina, USA

^f^ Department of Biostatistics, Harvard T.H. Chan School of Public Health, Boston, Massachusetts, USA

^g^ Department of Epidemiology, Harvard T.H. Chan School of Public Health, Boston, Massachusetts, USA

^h^ Obstetrics and Gynecology, Massachusetts General Hospital, Harvard Medical School, Boston, Massachusetts, USA

^*^ Corresponding author: 1835 SPH 1, 1415 Washington Heights, Ann Arbor, MI USA 48109. Telephone: 734-764-7184. Email: [meekerj@umich.edu](mailto:meekerj@umich.edu)

**Summary**

The supplemental tables below are the regression coefficients and 95% confidence intervals of which we derived our percent change in PFR concentrations for figures 1-4.

Supplemental Table 1: Regression coefficients (95% CI) for reported use of personal care products (PCP) and PFR metabolites within 24 hours of urine specimen collection for 230 women

|  |  | PFR Metabolites ^a^ | | | | | | | | | | | |
| --- | --- | --- | --- | --- | --- | --- | --- | --- | --- | --- | --- | --- | --- |
|  |  | BDCIPP | | | DPHP | | | ip-PPP | | | ∑PFR | | |
|  | n ^b^ | β | 95%CI | p | β | 95%CI | p | β | 95%CI | p | β | 95%CI | p |
| Deodorant | 378 | -0.06 | (-0.33, 0.20) | 0.64 | **0.25** | (0.05, 0.45) | 0.02 | **0.23** | (0.01, 0.44) | 0.04 | 0.18 | (-0.06, 0.43) | 0.14 |
| Shampoo | 367 | 0.10 | (-0.15, 0.35) | 0.43 | 0.02 | (-0.17, 0.21) | 0.85 | 0.02 | (-0.18, 0.23) | 0.81 | 0.12 | (-0.11, 0.36) | 0.31 |
| Toothpaste | 364 | 0.11 | (-1.14, 1.37) | 0.86 | 0.39 | (-0.58, 1.36) | 0.43 | 0.59 | (-0.43, 1.60) | 0.25 | 0.40 | (-1.39, 2.20) | 0.66 |
| Conditioner | 323 | -0.12 | (-0.35, 0.12) | 0.33 | 0.03 | (-0.15, 0.21) | 0.75 | **0.19** | (0.01, 0.38) | 0.04 | 0.05 | (-0.17, 0.26) | 0.67 |
| Bar Soap | 321 | -0.22 | (-0.46, 0.03) | 0.08 | -0.01 | (-0.20, 0.17) | 0.89 | 0.01 | (-0.19, 0.20) | 0.94 | -0.18 | (-0.40, 0.04) | 0.10 |
| Hand/Body Lotion | 298 | -0.02 | (-0.24, 0.21) | 0.88 | 0.06 | (-0.17, 0.23) | 0.53 | 0.04 | (-0.14, 0.22) | 0.69 | -0.05 | (-0.25, 0.16) | 0.66 |
| Colored Cosmetics | 203 | -0.16 | (-0.38, 0.06) | 0.14 | **0.24** | (0.07, 0.40) | 0.01 | **0.23** | (0.05, 0.40) | 0.01 | 0.13 | (-0.07, 0.33) | 0.21 |
| Face Moisturizer | 146 | 0.12 | (-0.37, 0.60) | 0.63 | **0.47** | (0.14, 0.80) | 0.01 | -0.03 | (-0.37, 0.31) | 0.87 | 0.21 | (-0.18, 0.60) | 0.29 |
| Hair Spray/Gel | 134 | -0.07 | (-0.31, 0.17) | 0.57 | 0.13 | (-0.06, 0.31) | 0.18 | 0.19 | (-0.001, 0.39) | 0.05 | 0.004 | (-0.21, 0.22) | 0.97 |
| Liquid Soap | 128 | 0.15 | (-0.14, 0.45) | 0.30 | 0.04 | (-0.19, 0.27) | 0.72 | 0.02 | (-0.24, 0.27) | 0.91 | 0.15 | (-0.13, 0.43) | 0.28 |
| Mouthwash | 115 | -0.03 | (-0.31, 0.24) | 0.82 | -0.06 | (-0.27, 0.15) | 0.57 | 0.08 | (-0.14, 0.30) | 0.46 | 0.09 | (-0.16, 0.35) | 0.47 |
| Cologne/Perfume | 114 | -0.08 | (-0.34, 0.17) | 0.53 | 0.16 | (-0.03, 0.36) | 0.10 | 0.17 | (-0.04, 0.37) | 0.11 | 0.12 | (-0.12, 0.35) | 0.33 |
| Other Toiletries | 94 | -0.18 | (-0.62, 0.25) | 0.41 | 0.02 | (-0.28, 0.33) | 0.88 | -0.14 | (-0.45, 0.16) | 0.35 | -0.23 | (-0.60, 0.13) | 0.21 |
| Other Hair Products | 92 | 0.12 | (-0.16, 0.40) | 0.39 | 0.17 | (-0.05, 0.38) | 0.13 | **0.24** | (0.02, 0.46) | 0.04 | 0.13 | (-0.11, 0.37) | 0.30 |
| Hand Sanitizer | 80 | -0.29 | (-0.59, 0.01) | 0.06 | 0.05 | (-0.18, 0.28**)** | 0.67 | 0.06 | (-0.18, 0.30) | 0.64 | -0.005 | (-0.26, 0.25) | 0.97 |
| Shaving Cream | 76 | 0.12 | (-0.19, 0.44) | 0.44 | 0.06 | (-0.18, 0.30) | 0.61 | -0.02 | (-0.27, 0.23) | 0.86 | -0.23 | (-0.50, 0.05) | 0.10 |
| Suntan/Block Lotion | 49 | 0.02 | (-0.33, 0.36) | 0.92 | 0.16 | (-0.11, 0.42) | 0.25 | 0.13 | (-0.15, 0.41) | 0.37 | 0.15 | (-0.17, 0.47) | 0.35 |
| Nail Polish Remover | 23 | 0.28 | (-0.26, 0.81) | 0.31 | **0.57** | (0.19, 0.95) | 0.004 | 0.05 | (-0.33, 0.43) | 0.81 | 0.28 | (-0.13, 0.70) | 0.17 |
| Nail Polish | 21 | -0.41 | (-0.89, 0.07) | 0.10 | **0.85** | (0.48,1.21) | <0.0001 | 0.19 | (-0.20, 0.59) | 0.33 | 0.42 | (-0.06, 0.81) | 0.09 |

^a^ Natural log transformation; ^b^ Reported ‘yes’ to PCP use within 24 hr. of PFR sample; Models adjusted for Specific gravity (SG), age, BMI, race (other/white), smoking status (never/ever), education & season; Reported use of PCP n <5 were not included in analysis

Supplemental Table 2: Regression coefficients (95% CI) for reported use of personal care products (PCP) and PFR metabolites within 24 hours of urine specimen collection for 229 men

|  |  | PFR Metabolites ^a^ | | | | | | | | | | | | | |
| --- | --- | --- | --- | --- | --- | --- | --- | --- | --- | --- | --- | --- | --- | --- | --- |
|  |  | BDCIPP | | | | DPHP | | | | ip-PPP | | | ∑PFR | | |
|  | n ^b^ | β | 95%CI | p | β | | 95%CI | p | β | | 95%CI | p | β | 95%CI | p |
| Deodorant | 211 | -0.10 | (-0.53, -0.33) | 0.65 | -0.21 | | (-0.52, 0.09) | 0.16 | 0.02 | | (-0.30, 0.35) | 0.90 | -0.15 | (-0.42, 0.11) | 0.25 |
| Shampoo | 204 | -0.09 | (-0.48, -0.30) | 0.64 | -0.09 | | (-0.37, 0.19) | 0.53 | 0.21 | | (-0.09, 0.50) | 0.17 | -0.06 | (-0.30, 0.19) | 0.65 |
| Toothpaste | 190 | **-**0.11 | (-1.09, 1.31) | 0.86 | -0.17 | | (-1.04, 0.70) | 0.70 | 0.25 | | (-0.70, 1.19) | 0.60 | -0.08 | (-0.85, 0.70) | 0.74 |
| Bar Soap | 177 | 0.04 | (-0.29, 0.38) | 0.79 | -0.13 | | (-0.37, 0.11) | 0.28 | 0.11 | | (-0.15, 0.36) | 0.40 | -0.04 | (-0.25, 0.17) | 0.70 |
| Shaving Cream | 87 | -0.10 | (-0.42, 0.22) | 0.53 | -0.15 | | (-0.37, 0.08) | 0.21 | 0.01 | | (-0.24,0.26) | 0.94 | -0.10 | (-0.30, 0.10) | 0.33 |
| Mouthwash | 79 | 0.23 | (-0.09, 0.55) | 0.16 | **0.27** | | (0.03, 0.49) | 0.03 | 0.15 | | (-0.10, 0.39) | 0.24 | **0.27** | (0.08, 0.48) | 0.01 |
| Hair Spray/Gel | 74 | 0.06 | (-0.28, 0.39) | 0.74 | 0.15 | | (-0.09, 0.39) | 0.22 | 0.15 | | (-0.10, 0.41) | 0.24 | 0.18 | (-0.03, 0.38) | 0.09 |
| Hand/Body Lotion | 63 | 0.04 | (-0.31, 0.39) | 0.82 | -0.17 | | (-0.42, 0.08) | 0.19 | -0.10 | | (-0.37, 0.17) | 0.47 | -0.07 | (-0.29, 0.15) | 0.52 |
| Liquid Soap | 59 | 0.23 | (-0.20, 0.66) | 0.28 | -0.12 | | (-0.45, 0.22) | 0.49 | -0.26 | | (-0.58, 0.07) | 0.12 | -0.01 | (-0.29, 0.28) | 0.97 |
| Hand Sanitizer | 54 | -0.12 | (-0.50, 0.26) | 0.51 | -0.08 | | (-0.35, 0.20) | 0.58 | -0.22 | | (-0.52, 0.07) | 0.14 | -0.14 | (-0.38, 0.11) | 0.26 |
| Cologne/Perfume | 41 | 0.29 | (-0.12, 0.70) | 0.17 | -0.04 | | (-0.34, 0.25) | 0.78 | 0.11 | | (-0.21, 0.42) | 0.50 | 0.06 | (-0.20, 0.32) | 0.63 |
| Conditioner | 40 | -0.04 | (-0.46, 0.38) | 0.85 | -0.08 | | (-0.38, 0.22) | 0.59 | 0.08 | | (-0.24, 0.40) | 0.62 | -0.08 | (-0.34, 0.18) | 0.55 |
| Face Moisturizer | 18 | -0.03 | (-0.72, 0.67) | 0.94 | -0.06 | | (-0.47, 0.36) | 0.78 | 0.005 | | (-0.50, 0.51) | 0.99 | 0.10 | (-0.30, 0.51) | 0.60 |
| Aftershave | 16 | 0.07 | (-0.52, 0.66) | 0.81 | 0.09 | | (-0.34, 0.51) | 0.69 | -0.42 | | (-0.87, 0.04) | 0.07 | 0.08 | (-0.29, 0.46) | 0.66 |
| Other Hair Products | 16 | 0.06 | (-0.54, 0.66) | 0.85 | 0.13 | | (-0.30, 0.57) | 0.53 | -0.12 | | (-0.59, 0.34) | 0.60 | 0.03 | (-0.35, 0.41) | 0.87 |
| Other Toiletries | 14 | -0.06 | (-0.86, 0.75) | 0.89 | -0.07 | | (-0.57, 0.43) | 0.78 | 0.08 | | (-0.53, 0.69) | 0.78 | -0.11 | (-0.58, 0.36) | 0.62 |
| Suntan/Block Lotion | 7 | 0.26 | (-0.60, 1.12) | 0.56 | -0.38 | | (-1.00, 0.24) | 0.22 | -0.43 | | (-1.10, 0.24) | 0.20 | -0.01 | (-0.56, 0.54) | 0.98 |

^a^ Natural log transformation; ^b^ Reported ‘yes’ to PCP use within 24hrs. of urine sample; Models adjusted for Specific gravity (SG), age, BMI, race (other/white), smoking status (never/ever), education & season; Reported use of PCP n <5 were not included in analysis

Supplemental Table 3: Regression coefficients (95% CI) for reported use of household products (HP) and PFR metabolites within 24 hours of urine specimen collection for 230 women (n=479)

|  |  | PFR Metabolites ^a^ | | | | | | | | | | | |
| --- | --- | --- | --- | --- | --- | --- | --- | --- | --- | --- | --- | --- | --- |
|  |  | BDCIPP | | | DPHP | | | ip-PPP | | | ∑PFR | | |
|  | n ^b^ | β | 95%CI | p | β | 95%CI | p | β | 95%CI | p | β | 95%CI | p |
| Laundry Detergent | 147 | -0.04 | (-0.26, 0.18) | 0.74 | -0.12 | (-0.29, 0.05) | 0.17 | 0.01 | (-0.17, 0.19) | 0.92 | -0.11 | (-0.32, 0.10) | 0.31 |
| Hand Dishwashing Liq. ^c^ | 123 | 0.002 | (-0.40, 0.40) | 0.99 | **-0.31** | (-0.59, -0.03) | 0.03 | 0.01 | (-0.27, 0.29) | 0.96 | -0.08 | (-0.40, 0.24) | 0.63 |
| Cleaners | 99 | -0.04 | (-0.31, 0.23) | 0.78 | -0.06 | (-0.26, 0.15) | 0.57 | 0.06 | (-0.15, 0.28) | 0.57 | 0.03 | (-0.21, 0.27) | 0.79 |
| Fabric Softener | 63 | -0.22 | (-0.54, 0.09) | 0.16 | -0.04 | (-0.28, 0.20) | 0.73 | -0.13 | (-0.38, 0.13) | 0.32 | -0.23 | (-0.52, 0.05) | 0.11 |
| Vinyl Gloves | 28 | 0.28 | (-0.22, 0.79) | 0.27 | 0.12 | (-0.26, 0.50) | 0.54 | 0.28 | (-0.12, 0.68) | 0.17 | 0.34 | (-0.11, 0.80) | 0.14 |
| Furniture Polish | 9 | 0.11 | (-0.65, 0.86) | 0.78 | -0.11 | (-0.69, 0.47) | 0.72 | -0.19 | (-0.80, 0.43) | 0.55 | -0.14 | (-0.78, 0.50) | 0.66 |
| Vinyl Boots | 8 | 0.15 | (-0.64, 0.95) | 0.70 | -0.15 | (-0.77, 0.46) | 0.62 | -0.31 | (-0.95, 0.34) | 0.35 | 0.05 | (-0.75, 0.86) | 0.90 |

^a^ natural log transformation; ^b^ Reported ‘yes’ to HP within 24hrs. of urine sample; ^c^ n=183; Models adjusted for Specific gravity (SG), age, BMI, race (other/white), smoking status (never/ever), education & season; Reported use of HP n <5 were not included in analysis

|  |  | PFR Metabolites ^a^ | | | | | | | | | | | | |
| --- | --- | --- | --- | --- | --- | --- | --- | --- | --- | --- | --- | --- | --- | --- |
|  |  | BDCIPP | | | DPHP | | | ip-PPP | | | ∑PFR | | | |
|  | n ^b^ | β | 95%CI | p | β | 95%CI | p | β | 95%CI | p | | β | 95%CI | p |
| Hand Dishwashing Liq. ^c^ | 59 | 0.23 | (-0.32, 0.78) | 0.39 | 0.003 | (-0.32, 0.33) | 0.98 | 0.08 | (-0.32, 0.48) | 0.68 | | 0.06 | (-0.26, 0.38) | 0.69 |
| Cleaners | 38 | 0.07 | (-0.33, 0.48) | 0.71 | 0.13 | (-0.16, 0.42) | 0.39 | 0.19 | (-0.12, 0.50) | 0.23 | | 0.14 | (-0.12, 0.39) | 0.29 |
| Laundry Detergent ^d^ | 22 | 0.27 | (-0.24, 0.77) | 0.30 | -0.27 | (-0.64, 0.10) | 0.15 | -0.02 | (-0.41, 0.38) | 0.94 | | -0.02 | (-0.35, 0.30) | 0.90 |
| Vinyl Gloves ^d^ | 9 | 0.65 | (-0.13, 1.42) | 0.10 | **0.65** | (0.09, 1.20) | 0.02 | 0.25 | (-0.35, 0.85) | 0.41 | | **0.52** | (0.03, 1.01) | 0.04 |
| Fabric Softener | 8 | -0.56 | (-1.35, 0.22) | 0.16 | -0.47 | (-1.05, 0.11) | 0.11 | -0.15 | (-0.78, 0.47) | 0.63 | | -0.42 | (-0.93, 0.09) | 0.10 |
| Paint/Solvent ^c^ | 5 | -0.16 | (-1.31, 0.99) | 0.77 | -0.27 | (-0.99, 0.46) | 0.45 | -0.49 | (-1.34, 0.36) | 0.25 | | -0.44 | (-1.08, 0.21) | 0.17 |

Supplemental Table 4: Regression coefficients (95% CI) for reported use of household products (HP) and PFR metabolites within 24 hours of urine specimen collection for 229 men (n=248)

^a^ natural log transformation; ^b^ n= reported using household products within 24hrs. of urine sample; ^c^ n=108; ^d^ n=247; Models adjusted for Specific gravity (SG), age, BMI, race (other/white), smoking status (never/ever), education & season; Reported use of HP n <5 were not included in analysis

Supplemental Table 5: Adjusted percent change (95% CI) for reported use of personal care products (PCP) and PFR metabolites within 24 hours of urine specimen collection for 230 women

|  |  | PFR Metabolites | | | | | | | | | | | | | | |  |
| --- | --- | --- | --- | --- | --- | --- | --- | --- | --- | --- | --- | --- | --- | --- | --- | --- | --- |
|  |  | BDCIPP | | | DPHP | | | | ip-PPP | | | | ∑PFR | | | |  |
|  | n ^a^ | %  change | 95%CI | p | | % change | 95%CI | p | | %  change | 95%CI | p | | %  change | 95%CI | p | |
| Deodorant | 378 | -6 | (-28, 22) | 0.64 | | **28** | (5, 57) | 0.02 | | **26** | (1, 55) | 0.04 | | 20 | (-6, 54) | 0.14 | |
| Shampoo | 367 | 11 | (-14, 42) | 0.43 | | 2 | (-16, 23) | 0.85 | | 2 | (-16, 26) | 0.81 | | 13 | (-10, 43) | 0.31 | |
| Toothpaste | 364 | 12 | (-68, 294) | 0.86 | | 48 | (-44, 290) | 0.43 | | 80 | (-35, 395) | 0.25 | | 49 | (-75, 803) | 0.66 | |
| Conditioner | 323 | -11 | (-30, 13) | 0.33 | | 3 | (-14, 23) | 0.75 | | **21** | (1, 46) | 0.04 | | 5 | (-16, 30) | 0.67 | |
| Bar Soap | 321 | -20 | (-37, 3) | 0.08 | | -1 | (-18, 19) | 0.89 | | 1 | (-17, 22) | 0.94 | | -16 | (-33, 4) | 0.10 | |
| Hand/Body Lotion | 298 | -2 | (-21, 23) | 0.88 | | 6 | (-16, 26) | 0.53 | | 4 | (-13, 25) | 0.69 | | -5 | (-22, 17) | 0.66 | |
| Colored Cosmetics | 203 | -15 | (-32, 6) | 0.14 | | **27** | (7, 49) | 0.01 | | **26** | (5, 49) | 0.01 | | 14 | (-7, 39) | 0.21 | |
| Face Moisturizer | 146 | 13 | (-31, 82) | 0.63 | | **60** | (15, 123) | 0.01 | | -3 | (-31, 36) | 0.87 | | 23 | (-16, 82) | 0.29 | |
| Hair Spray/Gel | 134 | -7 | (-27, 19) | 0.57 | | 14 | (6, 36) | 0.18 | | 21 | (-0.01, 48) | 0.05 | | 0.01 | (-19, 25) | 0.97 | |
| Liquid Soap | 128 | 16 | (-13, 57) | 0.30 | | 4 | (-17, 31) | 0.72 | | 2 | (-21, 31) | 0.91 | | 16 | (-12, 54) | 0.28 | |
| Mouthwash | 115 | -3 | (-27, 27) | 0.82 | | -6 | (-24, 16) | 0.57 | | 8 | (-13, 35) | 0.46 | | 9 | (-15, 42) | 0.47 | |
| Cologne/Perfume | 114 | -8 | (-29, 19) | 0.53 | | 17 | (-3, 43) | 0.10 | | 19 | (-4, 45) | 0.11 | | 13 | (-11, 42) | 0.33 | |
| Other Toiletries | 94 | -16 | (-46, 28) | 0.41 | | 2 | (-24, 39) | 0.88 | | -13 | (-36, 17) | 0.35 | | -21 | (-45, 14) | 0.21 | |
| Other Hair Products | 92 | 13 | (-15, 49) | 0.39 | | 19 | (-5, 46) | 0.13 | | **27** | (2, 58) | 0.04 | | 14 | (-10, 45) | 0.30 | |
| Hand Sanitizer | 80 | -25 | (-45, 1) | 0.06 | | 5 | (-16, 32**)** | 0.67 | | 6 | (-16, 35) | 0.64 | | -0.1 | (-23, 28) | 0.97 | |
| Shaving Cream | 76 | 13 | (-17, 55) | 0.44 | | 6 | (-16, 35) | 0.61 | | -2 | (-24, 26) | 0.86 | | -21 | (-39, 5) | 0.10 | |
| Suntan/Block Lotion | 49 | 02 | (-28, 43) | 0.92 | | 17 | (-10, 52) | 0.25 | | 14 | (-14, 51) | 0.37 | | 16 | (-16, 60) | 0.35 | |
| Nail Polish Remover | 23 | 32 | (-23, 125) | 0.31 | | **77** | (21, 159) | 0.004 | | 5 | (-28, 54) | 0.81 | | 32 | (-12, 101) | 0.17 | |
| Nail Polish | 21 | -34 | (-59, 7) | 0.10 | | **134** | (62, 235) | <0.0001 | | 21 | (18, 80) | 0.33 | | 52 | (-6, 125) | 0.09 | |

^a^ Reported ‘yes’ to PCP use within 24 hr. of PFR sample; Models adjusted for Specific gravity (SG), age, BMI, race (other/white), smoking status (never/ever), education & season; Reported use of PCP n <5 were not included in analysis

Supplemental Table 6: Adjusted percent change (95% CI) for reported use of personal care products (PCP) and PFR metabolites within 24 hours of urine specimen collection for 229 men

|  |  | PFR Metabolites | | | | | | | | | | | | | |
| --- | --- | --- | --- | --- | --- | --- | --- | --- | --- | --- | --- | --- | --- | --- | --- |
|  |  | BDCIPP | | | | DPHP | | | ip-PPP | | | ∑PFR | | | |
|  | n ^a^ | % change | 95%CI | p | %  change | | 95%CI | p | %  change | 95%CI | p | | %  change | 95%CI | p |
| Deodorant | 211 | -10 | (-41, 28) | 0.65 | -19 | | (-41, 9) | 0.16 | 2 | (-26, 42) | 0.90 | | -14 | (-34, 12) | 0.25 |
| Shampoo | 204 | -9 | (-38, 26) | 0.64 | -9 | | (-31, 21) | 0.53 | 23 | (-9, 65) | 0.17 | | -6 | (-26, 21) | 0.65 |
| Toothpaste | 190 | -10 | (-66, 271) | 0.86 | -16 | | (-65, 101) | 0.70 | 28 | (-50, 229) | 0.60 | | -8 | (-57, 101) | 0.74 |
| Bar Soap | 177 | 4 | (-25, 46) | 0.79 | -12 | | (-31, 12) | 0.28 | 12 | (-14, 43) | 0.40 | | -4 | (-22, 19) | 0.70 |
| Shaving Cream | 87 | -10 | (-34, 25) | 0.53 | -14 | | (-31, 8) | 0.21 | 1 | (-21, 30) | 0.94 | | -10 | (-26, 11) | 0.33 |
| Mouthwash | 79 | 26 | (-9, 73) | 0.16 | **31** | | (3, 63) | 0.03 | 16 | (-10, 48) | 0.24 | | **31** | (8, 62) | 0.01 |
| Hair Spray/Gel | 74 | 6 | (-24, 48) | 0.74 | 16 | | (-9, 48) | 0.22 | 16 | (-10, 51) | 0.24 | | 20 | (-3, 46) | 0.09 |
| Hand/Body Lotion | 63 | 4 | (-27, 48) | 0.82 | -16 | | (-34, 8) | 0.19 | -10 | (-31, 19) | 0.47 | | -7 | (-25, 16) | 0.52 |
| Liquid Soap | 59 | 26 | (-18, 93) | 0.28 | -11 | | (-36, 25) | 0.49 | -23 | (-44, 7) | 0.12 | | -1 | (-25, 32) | 0.97 |
| Hand Sanitizer | 54 | -11 | (-39, 30) | 0.51 | -16 | | (-30, 22) | 0.58 | -20 | (-41, 7) | 0.14 | | -13 | (-32, 12) | 0.26 |
| Cologne/Perfume | 41 | 34 | (-11, 101) | 0.17 | -4 | | (-29, 28) | 0.78 | 12 | (-19, 52) | 0.50 | | 6 | (-18, 38) | 0.63 |
| Conditioner | 40 | -4 | (-37, 46) | 0.85 | -8 | | (-32, 25) | 0.59 | 8 | (-21, 49) | 0.62 | | -8 | (-29, 20) | 0.55 |
| Face Moisturizer | 18 | -3 | (-51, 95) | 0.94 | -6 | | (-37, 43) | 0.78 | 1 | (-39, 67) | 0.99 | | 11 | (-26, 67) | 0.60 |
| Aftershave | 16 | 7 | (-41, 93) | 0.81 | 9 | | (-29, 67) | 0.69 | -44 | (-58, 4) | 0.07 | | 8 | (-25, 58) | 0.66 |
| Other Hair Products | 16 | 6 | (-42, 93) | 0.85 | 14 | | (-26, 77) | 0.53 | -11 | (-45, 40) | 0.60 | | 3 | (-30, 51) | 0.87 |
| Other Toiletries | 14 | -6 | (-58, 112) | 0.89 | -7 | | (-43, 54) | 0.78 | 8 | (-41, 99) | 0.78 | | -10 | (-44, 43) | 0.62 |
| Suntan/Block Lotion | 7 | 30 | (-45, 206) | 0.56 | -32 | | (-63, 27) | 0.22 | -35 | (-67, 27) | 0.20 | | -1 | (-43, 72) | 0.98 |

^a^ Reported ‘yes’ to PCP use within 24hrs. of urine sample; Models adjusted for Specific gravity (SG), age, BMI, race (other/white), smoking status (never/ever), education & season; Reported use of PCP n <5 were not included in analysis

Supplemental Table 7: Adjusted percent change (95% CI) for reported use of household products (HP) and PFR metabolites within 24 hours of urine specimen collection for 230 women (n=479)

|  |  | PFR Metabolites ^a^ | | | | | | | | | | | |  |
| --- | --- | --- | --- | --- | --- | --- | --- | --- | --- | --- | --- | --- | --- | --- |
|  |  | BDCIPP | | | DPHP | | | ip-PPP | | | ∑PFR | | |  |
|  | n ^b^ | %  change | 95%CI | p | %  change | 95%CI | p | %  change | 95%CI | p | %  change | 95%CI | p | |
| Laundry Detergent | 147 | -4 | (-23, 20) | 0.74 | -10 | (-25, 5) | 0.17 | 1 | (-16, 21) | 0.92 | -10 | (-27, 11) | 0.31 | |
| Hand Dishwashing Liq. ^c^ | 123 | 0.20 | (-33, 49) | 0.99 | **-27** | (-45, -3) | 0.03 | 1 | (-24, 34) | 0.96 | -8 | (-33, 27) | 0.63 | |
| Cleaners | 99 | -3 | (-27, 26) | 0.78 | -6 | (-23, 16) | 0.57 | 6 | (-14, 32) | 0.57 | 3 | (-19, 31) | 0.79 | |
| Fabric Softener | 63 | -20 | (-42, 9) | 0.16 | -6 | (-24, 22) | 0.73 | -12 | (-32, 14) | 0.32 | -21 | (-41, 5) | 0.11 | |
| Vinyl Gloves | 28 | 32 | (-20, 120) | 0.27 | 13 | (-23, 65) | 0.54 | 32 | (-11, 97) | 0.17 | 40 | (-10, 123) | 0.14 | |
| Furniture Polish | 9 | 12 | (-48, 136) | 0.78 | -10 | (-50, 60) | 0.72 | -18 | (-55, 54) | 0.55 | -13 | (-54, 65) | 0.66 | |
| Vinyl Boots | 8 | 16 | (-47, 159) | 0.70 | -14 | (-54, 58) | 0.62 | -27 | (-61, 40) | 0.35 | 5 | (-53, 136) | 0.90 | |

^a^ Reported ‘yes’ to HP within 24hrs. of urine sample; ^b^ n=183; Models adjusted for Specific gravity (SG), age, BMI, race (other/white), smoking status (never/ever), education & season; Reported use of HP n <5 were not included in analysis

Supplemental Table 8: Adjusted percent change (95% CI) for reported use of household products (HP) and PFR metabolites within 24 hours of urine specimen collection for 229 men (n=248)

|  |  | PFR Metabolites ^a^ | | | | | | | | | | | |
| --- | --- | --- | --- | --- | --- | --- | --- | --- | --- | --- | --- | --- | --- |
|  |  | BDCIPP | | | DPHP | | | ip-PPP | | | ∑PFR | | |
|  | n ^a^ | % change | 95%CI | p | % change | 95%CI | p | % change | 95%CI | p | % change | 95%CI | p |
| Hand Dishwashing Liq. ^b^ | 59 | 26 | (-27, 118) | 0.39 | 0 | (-27, 39) | 0.98 | 8 | (-27, 62) | 0.68 | 6 | (-23, 46) | 0.69 |
| Cleaners | 38 | 7 | (-28, 62) | 0.71 | 14 | (-15, 52) | 0.39 | 12 | (-11, 65) | 0.23 | 15 | (-11, 48) | 0.29 |
| Laundry Detergent ^c^ | 22 | 31 | (-21, 116) | 0.30 | -24 | (-47, 11) | 0.15 | -2 | (-34, 46) | 0.94 | -2 | (-30, 35) | 0.90 |
| Vinyl Gloves ^c^ | 9 | 92 | (-12, 314) | 0.10 | **92** | (9, 232) | 0.02 | 28 | (-30, 134) | 0.41 | **68** | (3, 175) | 0.04 |
| Fabric Softener | 8 | 57 | (-74, 25) | 0.16 | 63 | (-65, 12) | 0.11 | -14 | (-54, 60) | 0.63 | -34 | (-61, 9) | 0.10 |
| Paint/Solvent ^b^ | 5 | 85 | (-73, 169) | 0.77 | -23 | (-63, 58) | 0.45 | -39 | (-74, 43) | 0.25 | -36 | (-66, 23) | 0.17 |

^a^ n= reported using household products within 24hrs. of urine sample; ^b^ n=108; ^c^ n=247; Models adjusted for Specific gravity (SG), age, BMI, race (other/white), smoking status (never/ever), education & season; Reported use of HP n <5 were not included in analysis
